# Incipient Bulk Polycrystal Plasticity Observed by Synchrotron In-Situ Topotomography

**DOI:** 10.3390/ma11102018

**Published:** 2018-10-18

**Authors:** Henry Proudhon, Nicolas Guéninchault, Samuel Forest, Wolfgang Ludwig

**Affiliations:** 1 MINES ParisTech, PSL Research University, MAT—Centre des Matériaux, CNRS UMR 7633, BP 87 91003 Evry, France; ngueninchault@xnovotech.com (N.G.); samuel.forest@mines-paristech.fr (S.F.); 2 ESRF, The European Synchrotron, CS 40220, 38043 Grenoble, France; ludwig@esrf.fr; 3 INSA Lyon, MATEIS, University of Lyon, UMR 5510 CNRS, F-69621 Lyon, France

**Keywords:** polycrystal plasticity, X-ray diffraction imaging, topotomography, in situ experiment, finite element simulation, lattice curvature, rocking curve

## Abstract

**Abstract:**

In this paper, we present a comprehensive 4D study of the early stage of plastic deformation in a polycrystalline binary AlLi alloy. The entire microstructure is mapped with X-ray diffraction contrast tomography, and a set of bulk grains is further studied via X-ray topotomography during mechanical loading. The observed contrast is analyzed with respect to the slip system activation, and the evolution of the orientation spread is measured as a function of applied strain. The experimental observations are augmented by the mechanical response predicted by crystal plasticity finite element simulations to analyze the onset of plasticity in detail. Simulation results show a general agreement of the individual slip system activation during loading and that comparison with experiments at the length scale of the grains may be used to fine tune the constitutive model parameters.

**Dataset:**

10.5281/zenodo.1412401

## 1. Introduction

Determining microstructure-property relationships is an essential engineering problem and is directly linked to our ability to observe both the microstructure and the deformation/failure mechanisms concurrently. Electron back scattered diffraction (EBSD), which provides crystal orientation maps with sub-micrometer spatial resolution, remains a key tool, but is limited to the specimen surface [1]. To measure and interpret the strain field produced within individual grains, digital image correlation can be used provided a small-enough speckle can be produced at the specimen surface [2,3]. In this regard, subsequent analysis using numerical methods such as finite elements has also proved to be a powerful tool to interpret experimental results [4,5,6], but ultimately remains limited if the underlying material volume is not known [7]. In this paper, a new method combining in situ mechanical testing, three-dimensional (3D) bulk X-ray inspections and the crystal plasticity finite elements method (CPFEM) is used to study how plasticity proceeds in individual grains of a polycrystalline sample.

In the last 10 years, one particular focus of the 3D imaging community has been on obtaining reliable three-dimensional grain maps. Since most structural materials are polycrystalline and the mechanical properties are determined by their internal microstructure, this is a critical issue. For instance, considering slip transmission in crystal plasticity problems or small tortuous cracks evolving in a 3D grain network, it is a recurring challenge to assess the bulk mechanisms from surface observations only. Knowing how the different grains of the microstructure are arranged below the surface is therefore essential. Therefore, there has been considerable effort to develop characterization techniques at the meso-scale, which can image typically 1 mm${}^{3}$ of material with a spatial resolution in the order of the micrometer.

Among 3D characterization, an important distinction exists between destructive and non-destructive techniques. Serial sectioning relies on repeated 2D imaging (which may include several modalities) of individual slices where a thin layer of material is removed between each observation [8]. Considerable progress on that side has been made in the last decade, bringing high quality measurements in 3D of grain sizes and orientations, but also detailed grain shapes and grain boundary characteristics. The price to pay with serial sectioning remains, however, the destruction of the sample. In parallel, accessing crystallographic information in the bulk of polycrystalline specimens was subsequently achieved by using the high penetration power of hard X-rays and leveraging diffraction contrast. This led to the development of a variety of 3D X-ray diffraction techniques (3DXRD; see [9,10]) enabling the characterization of millimeter-sized specimens by tracking the diffraction of each individual crystal within the material volume while rotating the specimen. Among them, the near field variant called X-ray diffraction contrast tomography (DCT) uses an extended box beam to illuminate the specimen and allows for simultaneous reconstruction of both the sample microstructure visible in X-ray absorption contrast and the crystallographic grain microstructure as determined from the diffraction signals with a single tomographic scan provided the grains have a limited orientation spread [11,12,13]. The typical acquisition time is one hour on a high brilliance beam line such as ID11at ESRF, which makes it the fastest non-destructive grain mapping technique.

Non-destructive imaging allows one to observe both the microstructure and the deformation/failure mechanisms in situ (4D studies). However, resolving 3D grain shapes by near-field diffraction imaging requires reducing the sample to detector distance to a few millimeters, which has for a long time drastically limited mechanical 4D studies due to the space constraints. Recent progresses with mechanical stress rigs solved this issue and opened new perspectives to study the deformation and fracture of polycrystalline materials [14,15].

Another key challenge is to link 3D microstructure characterization tools with computational models in order to predict engineering mechanical properties. This can be done using synthetically-generated images, but it requires using sophisticated models to ensure that the microstructure is representative, in particular of the tail distributions (critical when looking at fracture processes) [16]. Another approach is to use as-measured 3D microstructures [17]. The major advantage of this route is directly comparing experiment and mechanical simulation at the grain scale. For mechanical problems, continuum crystal plasticity (either using the finite elements or the spectral method to solve the equilibrium) has proved to be a powerful tool to interpret experimental results obtained in the deformation of metallic polycrystals [4,18,19,20]. Large-scale 3D polycrystalline simulations can be performed with sufficient local discretization to predict the transgranular plastic strain fields. One of the issues of this type of models is the material parameters’ identification. This is mostly due to the fact that identification is classically done by minimizing a cost function versus macroscopic tests’ response, which makes the problem ill posed. Some recent attempts directly used local strain measurements in the identification dataset in order to identify material model parameters [21], but have remained limited to surface studies so far.

The mechanical behavior of polycrystalline alloys is especially important for structural materials. A few key examples are Al alloys used for transports in general, Ti alloys used in aerospace where specific strength and high performance is particularly needed or Ni base alloys when looking for very high temperature performances. In this regard, advancing our understanding on the deformation of such materials will lead to lighter, stronger and more reliable parts.

In this paper, we present a new 4D study, using a combination of X-ray diffraction contrast tomography, topotomography and phase contrast tomography to study polycrystal plasticity in an aluminum-lithium alloy. Experimental and simulation methods are detailed in Section 2. The main experimental results are then presented in Section 3.1 for the observed slip system activation and Section 3.2 for the measured crystal lattice orientation. Simulation results and comparison with the experiments are described in Section 3.3. In the Conclusion Section, the main results are summarized, and some future directions of research are suggested.

## 2. Materials and Methods

### 2.1. Experimental Setup for Topotomography

A plate made of Al-Li 2.5% wt purchased from MaTeck was rolled to 45% reduction and recrystallized 20 min at 530 ${}^{\circ }$C to tune the grain size at 100 μm. This step ensured the number of grains in the illuminated section was well below the DCT limit (typically a few thousands) and that the initial state was as defect free as possible to study the onset of plasticity. The material was then aged 4 h at 100 ${}^{\circ }$C to form very small Al${}_{3}$Li precipitates with an expected size of 8 nm [22]. At this size, the precipitates remain shearable by the dislocations, which will promote a planar and localized slip as the main deformation mechanism. Small dog-bone tomographic tension specimens (0.5 mm × 0.5 mm minimal cross-section) were cut using EDM in the middle of the plate for the experiment. A 8.5-mm radius in the gauge length produces a small stress concentration and ensures that the first plastic events will occur in the observed region.

The experiment was carried out at the material science beam line (ID11) at the ESRF. The X-ray beam was produced by an in-vacuum undulator and collimated to a size of about 0.7 × 0.7 mm by means of an X-ray transfocator [23]. The beam energy was set to 40 keV with a relative bandwidth of $3\times 10^{-3}$. The diffractometer installed on ID11 has been designed for this particular variant of diffraction imaging experiments, which requires the alignment of a scattering vector parallel to the tomographic rotation axis [24,25]. The scattering vector $\underline{G}\;$ is defined as $\underline{G}\;=\underline{K}\;-\underline{X}\;$ with $\underline{X}\;$ the incident wave vector and $\underline{K}\;$ the diffracted wave vector, both with a norm of 1/λ. Let us call (p,q,r) the components of the scattering vector ${\underline{G}\;}_{s}$ expressed in the sample coordinate system for a set of (hkl) planes:

(1) $${\underline{G}\;}_{s}=g^{-1}B\left(\begin{array}{c} h\\ k\\ l \end{array}\right)=\left(\begin{array}{c} p\\ q\\ r \end{array}\right)$$

where g is the orientation matrix of the crystal and B accounts for the lattice geometry (for cubic structures like aluminum, it reduces to the lattice parameter times the identity).

Following [26,27] and accounting for the four diffractometer rotation angles (Θ, ω, ϕ, χ), via the four associated rotation matrices (T, Ω, Φ, X), the Bragg diffraction condition in 3D can be written as:

(2) $$\frac{2\sin^{2}\left(\theta_{Bragg}\right)}{\lambda }=-{\left[T\Omega \Phi X\left(\begin{array}{c} p\\ q\\ r \end{array}\right)\right]}_{1}$$

where the rotation matrix X associated with the diffractometer angle χ should not be confused with the incident wave vector $\underline{X}\;$.

**Figure 1:**
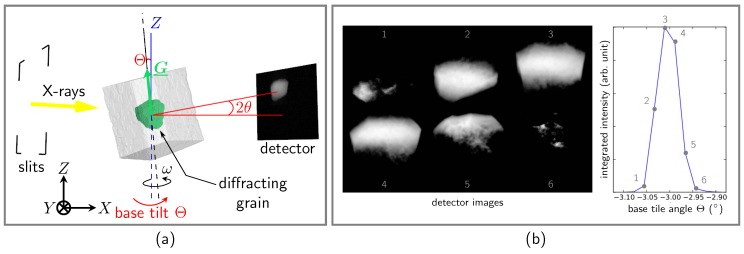
Schematics of the topotomographic alignment: (**a**) a scattering vector is put on the rotation axis (ω), and the whole setup is tilted by the nominal value $\Theta =\theta_{Bragg}$; (**b**) example of integrated detector intensity to build the rocking curve.

In a topotomographic experiment, the scattering vector is aligned with the rotation axis of the tomographic rotation stage ω (see Figure 1a). Circles χ and ϕ are used to set $\underline{G}\;$ parallel to the rotation axis ω (matrices X and Φ, respectively), while Θ is used to tilt the whole setup (including the tomographic rotation axis) around *Y* by the Bragg angle $\theta_{Bragg}$ (matrix T). For a known crystal structure and orientation (i.e., B and g are known), it is straightforward to rework Equation (2) to derive the two tilt rotation values (χ,ϕ):

(3) $$\begin{array}{cc} \chi & =\arctan\left(-\frac{p}{r}\right) \end{array}$$

(4) $$\begin{array}{cc} \phi & =\arctan\left(\frac{q}{-p\sin(\chi )+r\cos(\chi )}\right) \end{array}$$

Equation (2) is then fulfilled for all possible values of ω. Note that in the DCT case, T, Φ and X vanish, and the equation is only verified for 2 particular values, solutions of a quadratic equation in ω known as the rotating crystal problem [26].

It is important to understand clearly the difference between Θ and $\theta_{Bragg}$, as they differ in nature. Θ is the value of the tilt applied by the base tilt motor, whereas $\theta_{Bragg}$ is a material parameter associated with a given $d_{hkl}$ spacing and wavelength λ: $\theta_{Bragg}=\arcsin\lambda /2d$.

Now, in the ideal case of a perfect crystal and quasi-monochromatic plane wave illumination, the entire grain would diffract for the position of the base tilt $\Theta =\theta_{Bragg}$, and only a simple rotation around ω would be needed to collect the topotomographic dataset (see Figure 1a). This is in practice never the case, as the inner mosaicity of the grain and dispersion effects require rocking the base tilt (this is again a rotating crystal problem, which is covered in more detail in Section 2.5). A topotomographic (TT) dataset can therefore be seen as a collection of rocking curves and associated stacks of 3D projection data and can be used in two different ways. First, integrated projection topographs corresponding to projections of the entire crystal volume are obtained by summing the intensity over the base tilt Θ. Inspection of these topographs (see Figure 1b) allows for direct identification of the presence of crystalline defects such as slip bands. Second, the width of the rocking curve I=f(Θ) for a given ω position is a measure of the lattice rotation around the base tilt axis (*Y* here; see Figure 1c).

### 2.2. Details of the In Situ Experiment

**Figure 2:**
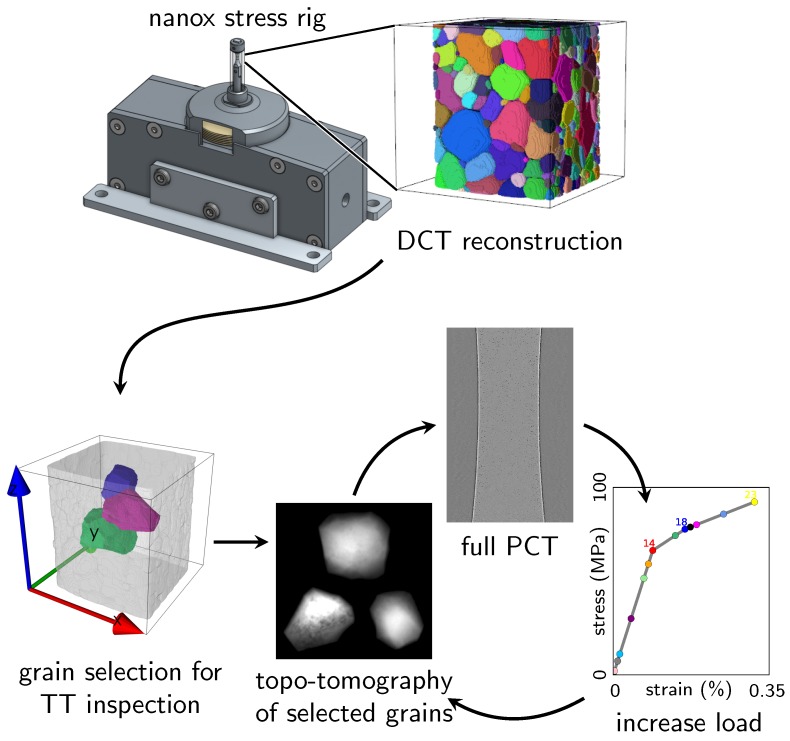
Sequence of the in situ topotomography experiment: the initial microstructure is characterized by DCT and analyzed after mounting the specimen into the Nanox device [15], then 23 complete sequences, each comprising three topotomography and one phase contrast tomography scan, have been recorded at increasing levels of load (the color code used in the tension curve is used later on to plot results at a given load level).

A small tomographic tension specimen was mounted in the Nanox stress rig, specifically designed to be compatible with both DCT and topotomography acquisition geometries [15]; see Figure 2, left. The machine has a very limited size and weight and, thanks to the load bearing quartz tube, allows $360^{\circ }$ visibility in the DCT configuration with the detector as close as 3 mm to the rotation axis. Full visibility is also achieved in the TT configuration with $\theta_{Bragg}\leq 10^{\circ }$, with the detector as close as 10 mm from the rotation axis, for the complete range of motion of the two inner diffractometer circles ($\pm 20^{\circ }$ and $\pm 15^{\circ }$ for Φ and χ, respectively). For given values of $\theta_{Bragg}$, Φ and χ, it is possible to move the TT detector even closer, but this has to be checked manually.

**Figure 3:**
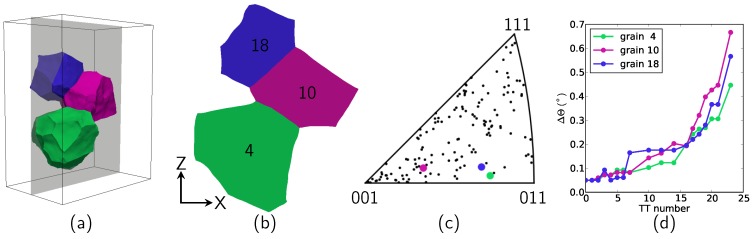
Details on the 3-grain cluster (**a**) 3D visualization of the grains (**b**); XZ slice through the 3 grains; (**c**) inverse pole figure of the gauge length with the 3 grain orientations of interest highlighted; (**d**) Θ integration range automatically determined at each loading step.

With the specimen inside the stress rig, it is possible to analyze the initial undeformed bulk microstructure (positions, shapes and grain orientations) by a DCT scan. The DCT data are then processed to extract all grain orientations and positions within the gauge length. Later on, the DCT reconstruction is also used as input to perform 3D CPFEM calculations (see Section 2.3). For now, these data are used to select a series of grains for further analysis using topotomography imaging during mechanical loading. Here, the selection was based on the following criteria: (i) a low order reflection must be accessible (note that the two circles Φ, χ used for the topotomographic alignment only have a limited range of motion); (ii) the grain must be located in the bulk of the specimen; (iii) all the selected grains must form a small neighborhood. Grain Numbers 4, 10 and 18 (see Figure 3), located in the central region of the specimen, fit these constraints and were selected for the present study to carry out a series of topotomographic scans during a (interrupted) tensile test; see Figure 2, right.

At each of the 23 load steps, a phase contrast tomography of the gauge length (1.5 mm in height) is also recorded and used to extract macroscopic strain information. The essential information (grain orientation, aligned hkl reflection, Bragg angle, diffractometer angles) for each grain is reported in Table 1. Grains 10 and 18 have a similar orientation, as seen in Figure 3c).

DCT scans were composed of 3600 equally-spaced projections over 360${}^{\circ }$, recorded on a high-resolution detector with a transparent luminescent screen optically coupled by a 10× microscope objective to a 2048 × 2048 pixel ESRF Frelon camera, giving an effective pixel size of 1.4 μm. A 0.3-s exposure time has been used, resulting in an acquisition time of about 40 min. The same camera was used to record the PCT scans; the camera traveled back and forth along the *X*-axis from the DCT position (about 5 mm behind the specimen) to the PCT position, about 105 mm downstream. A second detector system with a 20× objective and an effective pixel size of 0.7 μm was used for topotomographic scan acquisition. The angular range of the rocking curve scans was automatically adjusted after each load increment in order to cover the entire width of the crystal reflection curve for any ω rotation position of the sample. Moreover, the X-ray flux density was further increased by focusing the beam on the area covered by the 3-grain cluster. A continuous motion acquisition procedure with a fixed integration range of $0.1^{\circ }$ and 0.5-s exposure time per image was used. Integration gaps caused by the readout time of the CCD detector could be eliminated by operating the system in frame transfer mode. In this mode, only half of the active area is available for image acquisition, whilst the other half is used for temporary storage and readout of the previous frame. This procedure was repeated every $4^{\circ }$, and a complete topotomographic acquisition comprising 90 such rocking scans per grain typically lasted from 10 min up to an hour as the Θ integration range increased during loading. In this experiment, the integration range determination after each load step has been automated by acquiring a coarse TT scan at two values of ω separated by $90^{\circ }$, post-processing the intensity in the image and taking the largest bounds of the two rocking curves, increased by a small amount not to miss any intensity. From the beginning to the end of the experiment, the integration range has been multiplied by a factor of 10 (from $0.05^{\circ }$ to $0.5^{\circ }$; see Figure 3d).

### 2.3. Crystal Plasticity Finite Element Simulations

A finite strain crystal plasticity model, fully described in [29], is used here to compute the mechanical response of the polycrystalline sample under tension. It is based on the multiplicative decomposition $\underset{∼}{F}=\underset{∼}{E}\underset{∼}{P}$ of the deformation gradient, $\underset{∼}{F}$, into an elastic part, $\underset{∼}{E},$ and a plastic part, $\underset{∼}{P}$. The multiplicative decomposition is associated with the definition of an intermediate configuration for which the elastic part of the deformation gradient is removed. The intermediate released configuration is uniquely determined up to a rigid body rotation, which is chosen such that the lattice orientation in the intermediate configuration is the same as the initial one. Mandel called it the isoclinic intermediate configuration [30]. As a result, lattice rotation and distortion during elastoplastic deformation are contained in the elastic deformation part $\underset{∼}{E}$. The transformation $\underset{∼}{E}$ has a pure rotation part ${\underset{∼}{R}}^{e}$ and a pure distortion part ${\underset{∼}{U}}^{e}$, which can be obtained by the polar decomposition:

(5) $$\underset{∼}{E}={\underset{∼}{R}}^{e}{\underset{∼}{U}}^{e}$$

Plastic deformation is the result of slip processes according to a collection of *N* slip systems, each one characterized by the slip direction ${\underline{m}\;}^{s}$ and the normal to the slip plane ${\underline{n}\;}^{s}$. Note that here, plastic slip is the only deformation mechanism considered. This has been double checked up to 10% strain by performing an in situ tensile test in a scanning electron microscope (not reported here for brevity). In other cases, mechanical twinning or grain boundary sliding may need to be considered. In the intermediate configuration, $\underset{∼}{P}$ verifies:

(6) $$\dot{\underset{∼}{P}}{\underset{∼}{P}}^{-1}=\sum_{s=1}^{N}{\dot{\gamma }}^{s}{\underline{m}\;}^{s}⊗{\underline{n}\;}^{s}$$

In order to analyze the microplastic behavior of the studied AlLi polycrystal, an elasto-visco-plastic crystal plasticity model was selected. Numerical computations were performed using the Z-set software (http://www.zset-software.com) (see [31]). The slip rate on a given slip system *s* depends, via a phenomenological power law with two parameters *K* and *n*, on how much the resolved shear stress $\tau^{s}$ exceeds the threshold $\tau_{0}+r^{s}$:

(7) $${\dot{\gamma }}^{s}={\langle \frac{|\tau^{s}|-\tau_{0}-r^{s}}{K}\rangle }^{n}\mathrm{sign}\left(\tau^{s}\right)$$

Here, $\tau_{0}$ is the critical resolved shear stress, and $r^{s}$, initially zero, increases with increasing plastic strain and hardens the system *s* through a non-linear isotropic Voce hardening rule, as developed in [32]:

(8) $$r^{s}=Q\sum_{r=1}^{N}h^{sr}\left(1-\exp\left(-bv^{s}\right)\right)$$

$v^{s}$ is the cumulative slip and $h^{sr}$ denotes the interaction matrix taking into account the relative influence of slip systems on each other. It includes the self and latent hardening, and only indirect and estimated quantitative information is available about the components of this matrix (see for instance [33,34]). *Q* and *b* are 2 material parameters to be determined.

Monotonic tensile tests were performed on five different macroscopic samples, at three strain rates, and a numerical optimization using Z-set implementation was performed to find a suitable parameter set $\left(\tau_{0},Q,b\right)$, as presented in Table 2. The yield stress of the material exhibits an inverse strain rate sensitivity, which prevented identifying the viscosity parameters *K* (not to be confused with the norm of the diffraction vector $\underline{K}\;$) and *n*; instead, sensible values for aluminum alloys have been used. Modeling this effect requires a more complex model, including dynamic strain aging as in [35]. For the interactions between dislocations, coefficients $h^{rs}$ from [36] have been used.

**Figure 4:**
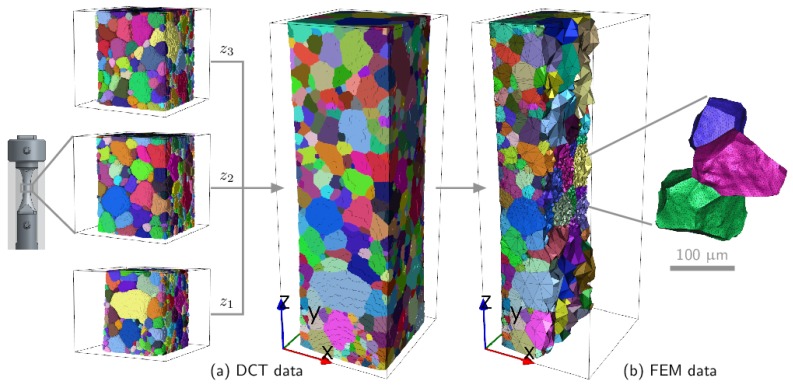
Comparison between the DCT data (**a**) and the mesh generated (**b**); the colors denote the grain numbers, which are consistent from the experiment to the simulation. Note the specimen shape with a radius in the gauge length as mentioned in Section 2.

The experimental grain map is used as input to produce a high fidelity digital clone of the specimen. The entire L=1.57 mm zone, where 3 DCT scans were merged, was used to ensure the boundary condition application is far enough from the grains of interest to avoid any boundary layer effect [37]. Details on how the mesh was produced can be found in [17]. The initial grain boundary surface generated contains a very large number of triangles, and an iterative decimation approach using an edge collapsing algorithm is applied. The surface mesh is filled with tetrahedra controlling the mesh density as a function of the euclidean distance *d* from the three-grain cluster. This allowed minimizing the computational cost while preserving a rich description of the mechanical fields in the region of interest. The final mesh is composed of 341,687 linear tetrahedra with a gradient in element size (the ratio between the maximum and minimum tetrahedron size is about 4000) visible in Figure 4.

Dirichlet boundary conditions ($u_{z}=0$ on the lower face and $u_{z}=15.7$ microns on the upper face for the final deformation step) were imposed to deform the specimen in tension up to 1% total strain in 100 steps. Suitable boundary conditions have been set on the lower surface of the sample to prevent any rigid body motion, and lateral surfaces were free of stress. The steps corresponding to the experimentally-measured strain (for instance 0.32% total strain) can be used for comparison.

### 2.4. Lattice Rotations

The continuum mechanical approach used here makes it possible to distinguish between the transformation of material and lattice directions. Material lines are made of material points that are subjected to the motion field u. In contrast, lattice directions are not material insofar as they are not necessarily made of the same material points (atoms) in the initial and current configurations due to the passing of dislocations, but keep the same crystallographic meaning. According to the concept of isoclinic configuration, lattice directions are unchanged from the initial to the intermediate configuration. Dislocations passing through a material volume element do not distort nor rotate the lattice, although material lines are sheared. According to the continuum theory of dislocations, statistically-stored dislocations accumulating in the material volume element affect material hardening, but do not change the element shape. Accordingly, an initial lattice direction ${\underline{d}\;}^{♯}$ is transformed into $\underline{d}\;$ by means of the elastic deformation:

(9) $$\underline{d}\;=\underset{∼}{E}.{\underline{d}\;}^{♯}$$

This important distinction allows one to precisely compute both the local rotation and distortion of the crystal lattice, which will be further used to derive the 3D rocking curve of a grain from its deformed state in the simulation (see Section 2.5).

### 2.5. Rocking Curves Simulations from CPFEM Data

As explained in Section 2.1, the rocking curve represents the intensity diffracted by the illuminated grain at a given Θ angle. As soon as the crystal deforms, the exact Bragg condition is violated, and the I(Θ) curve will widen. In crystal plasticity, geometrically necessary dislocations (GND) give rise to gradients of crystal orientation, leading to local modification of the Bragg condition. The problem is therefore to solve the 3D diffraction condition stated in Equation (2) for the angle Θ, with a locally deformed crystal lattice. In Equation (2), the values of (p,q,r), as well as $\theta_{Bragg}$ need to be updated for the new lattice geometry. It is therefore possible to use the mechanical fields computed in each element (namely $\underset{∼}{E}$ and ${\underset{∼}{R}}^{e}$) to evaluate locally the Bragg condition (for a given ω value) and to find the corresponding Θ. Building the volume weighted histogram for all elements within the grain will produce a simulated rocking curve (for this value of ω).

## 3. Results

### 3.1. Topography Results

X-ray topographs are 2D oblique projections of a crystal. At the onset of plasticity, slip system activity may modify the local Bragg condition within the grain and produce orientation contrasts on the detector. In this section, topographs over a full ω turn for three neighboring grains as a function of the load are analyzed.

As we shall see, the perturbations of the crystal lattice are localized within the slip plane and may only be visible at certain ω angles, called edge-on configuration, when the diffraction direction is contained in the plane (i.e., the tilted slip plane normal ${\underline{n}\;}_{t}$ is perpendicular to $\underline{K}\;$). Knowing the grain orientation and the tilt geometry, it is straightforward to obtain the two edge-on ω angles for a given slip plane by solving (here, we do not account for the base tilt, as both the left and right side of the equation would be equally affected):

(10) $$\left(\Omega .{\underline{n}\;}_{t}\right).\underline{K}\;=0\;\mathrm{with}\;\underline{K}\;=\frac{1}{\lambda }\left(\begin{array}{c} \cos(\theta_{Bragg})\\ 0\\ \sin(\theta_{Bragg}) \end{array}\right)$$

This means solving $\left[n_{t}\left[0\right]\cos\left(\omega \right)-n_{t}\left[1\right]\sin\left(\omega \right)\right]\cos\left(\theta \right)+n_{t}\left[2\right]\sin\left(\theta \right)=0$. The two ω values, for a given slip plane, are separated by close to 180${}^{\circ }$, a value depending on $\theta_{Bragg}$.

Table 3 gathers the ω values for the two slip systems with the highest Schmid factor in the 3three grains calculated with Equation (10). These values will be used to show the topographs in edge-on configuration where the contrast is expected to be maximal for a given slip plane. Note that the values for the two observed slip planes (not to be confused with the two values ($\omega_{1},\omega_{2}$) for a given slip plane) are exactly separated by 180 degrees. For Grains 4 and 18, the aligned reflection is (202). Rotating around this axis, the edge-on configurations for the slip planes with the two highest Schmid factors $\left(1\bar{1}1\right)$ and (111) are 180${}^{\circ }$ apart (they share the $\left[\bar{1}01\right]$ zone axis, which is perpendicular to the scattering vector). For Grain 10, the aligned reflection is (002); rotating around this axis, the edge-on configurations for the (111) and $\left(11\bar{1}\right)$ planes are also 180${}^{\circ }$ apart (they share the $\left[\bar{1}10\right]$ zone axis, which is perpendicular to the scattering vector).

The Schmid factor allows estimating the resolved shear stress and is here calculated in the single crystal approximation. It should be noted that it cannot rigorously be applied to the polycrystal case (this value neglects the effect of neighboring grains and any other heterogeneity), although it is very often used to predict slip system activation. It is thus interesting to see in this case how well this indicator performs. In contrast to the estimation using the Schmid factor, the full field finite element simulations performed in this work (see Section 3.3) take into account the interaction of individual grains with their neighbors. This results in strongly non-homogeneous fields of plastic strain and lattice rotation inside the grains. The advantage of the Schmid factor is clearly that it depends only on the crystal orientation (and is thus easily computed on the fly during this type of diffraction experiment). However, the material scientist doing an experiment may rely on a more detailed stress estimation, either using a supplementary far field detector and track the motion of diffraction spots [19] or inferring the stress tensor value from mean field or full field computations using the actual 3D microstructure, as done in the present work.

**Figure 5:**
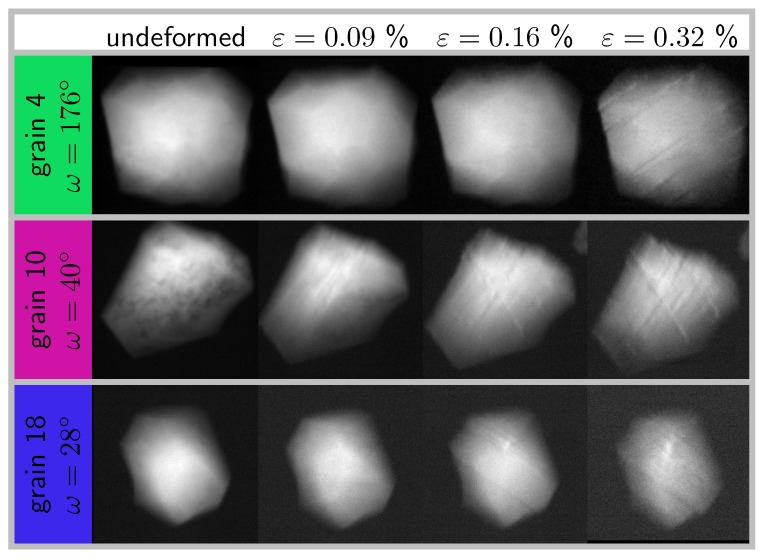
Topographs, integrated over Θ, in edge-on configuration for each grain of the cluster and different load levels; videos of the complete ω set are available as Supplementary Material for Grain 4 in the initial and deformed states.

Integrated topographs at the recorded ω closest to the edge-on values and for selected load levels are shown in Figure 5. Contrast forming bands within the grains are clearly visible and appear first for Grain 10, then Grain 18 and finally for Grain 4.

These sets of bands were parallel; most of the time, they extended through the whole grain, and their number increased as the deformation increased. Going through the whole projection set for each grain shows that two sets of bands were visible at different ω values. For Grain 10, the crystallographic configuration was such that both sets were visible at around $40^{\circ }$. This is, to the best of the author knowledge, the first in situ observation of bulk plasticity in a millimeter sized polycrystalline specimen. From there, the orientation of the bands, their number and location within the grain can be further studied.

**Figure 6:**
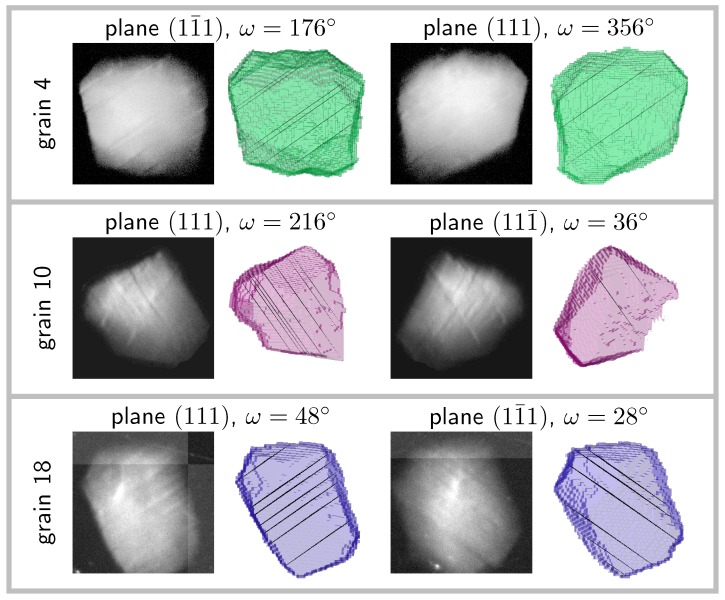
Identification of the bands as slip plane traces visible in the edge-on geometrical configuration in the topographs; here, the two observed active slip planes are shown for one of the two ω angles; the active slip plane locations within the grains have been measured manually and displayed in 3D.

Using the initial grain orientation and the tilt geometries for each aligned reflection, it is possible to correlate the angle of those bands to specific crystallographic planes (see Figure 6). For this, a 3D geometrical representation of the grain was built using the DCT reconstruction, and the relevant slip planes had been added inside according to the measured grain orientation (the open source library pymicro [38] was used to this end). The grain can be tilted in the topotomographic condition and rotated to the given edge-on omega angle. Using a parallel projection mode and setting the view in the diffracted beam direction $\underline{K}\;=\left(1,0,\tan\left(2\theta_{Bragg}\right)\right)$ produced the same conditions as when collecting the topographs. All the observed bands had been identified without any ambiguity as projections of the {111} planes; see Figure 6. In each case, the exact slip plane could be identified and further related to the Schmid factor. This demonstrates that the onset of plasticity, from the first slip band to the more advanced state where several slip systems are active, was indeed captured in situ during this experiment.

For Grains 4 and 18, the two observed slip planes correspond to the two highest Schmid factors (see Table 3), whereas for Grain 10, they correspond to the first and third highest Schmid factors. The slip system corresponding to the second highest Schmid was not observed to be active in this grain.

**Figure 7:**
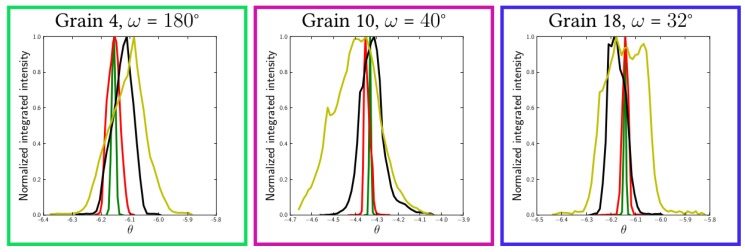
Evolution of the rocking curves for each grain of the cluster during in situ loading; the curve colors (green, red, black, yellow) refer to the load levels in Figure 5, respectively (undeformed, 0.09%, 0.16% and 0.32%).

Rocking curves are presented in Figure 7, for each grain at the strain levels and for the same ω values as in Figure 5. All three grains exhibited a consistent behavior, with a narrow curve at the beginning, which first shifted to the lower $|\theta_{Bragg}|$ values due to elastic loading (increase of the $d_{hkl}$ interplanar spacing) and then widened considerably when plasticity took place.

### 3.2. 3D Rocking Curves’ Results

As grains are aligned in a topotomographic sense, it is possible to measure rocking curves at every ω position. This measure is therefore sensitive not only to the amount of curvature of a crystal, but also to the orientation of this curvature in real space. To quantify the intragranular orientation spread revealed by a rocking curve, we introduced the width of the rocking curve at 10% of the peak of the normalized intensity, denoted as full width of the effective misorientation (FWEM). Although the effective misorientation describes the change in Bragg condition due to both orientation and lattice spacing variations, in practice, the orientation effect is largely predominant. Therefore, this value is a direct (qualitative) measure of the orientation spread, around the axis defined by the base tilt.

**Figure 8:**
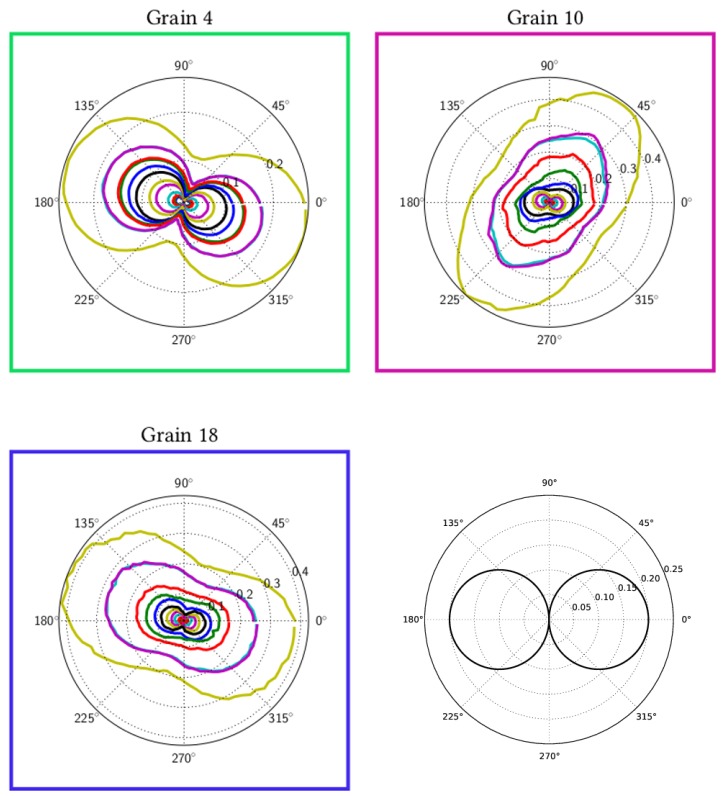
In situ FWEM as a function of ω, for each grain of the cluster (the color code matches the one used for the tensile curve in Figure 2) and example of the FWEM in the case of a crystal ideally bent around a single axis by $0.2^{\circ }$ (bottom right).

The FWEM was measured every $4^{\circ }$ (for each ω position) and is plotted at all different load levels in Figure 8 to observe its evolution with increasing plasticity. An interesting dumbbell-shaped curve was consistently obtained for the three grains. The curve widened in a preferential direction linked to the active slip systems within the grain. One can observe that the FWEM was similar for Grains 4 and 18, which have the same combination of active slip systems. The orientation of the curves for Grain 10 was different and exhibited a clear reorientation of the preferential direction towards the end of the loading sequence. This may be linked to changes in the relative activity of the dominant slip system(s) during deformation, but would require further analysis.

The shape of the curve can be understood more clearly considering the idealized case of a strain-free crystal bent by an amount ΔΘ by geometrically necessary dislocations. In the kinetic approximation, this configuration would produce a figure made of two tangent circles giving a very limited FWEM in the bending axis direction (almost no lattice rotation) and of exactly ΔΘ perpendicular to it. The FWEM can be seen as the limit of the Bragg condition when rocking the base tilt Θ and can be computed using the rotating crystal equation solving for Θ for a known ω (see Figure 8, bottom right).

### 3.3. Comparison with CPFEM Simulations

The first comparison is the slip activity computed with the numerical model (seen Section 2.3) for each grain with the activity visible on the topographs for a particular ω position (see Section 3.1). The CPFEM simulations give access to the active slip systems as opposed to the slip planes only, which were identified experimentally on the topograph. The slip activity was captured by averaging the amount of slip for a given slip system within the grain and can be compared to the qualitative information obtained on the topographs (intensity of the contrast and number of bands).

**Figure 9:**
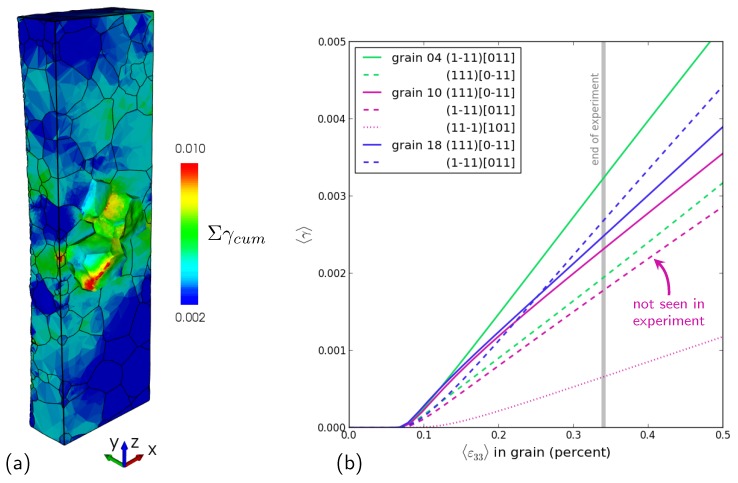
Predicted plastic activity: (**a**) view of the interior of the specimen showing the accumulated plasticity at the load level corresponding to the end of the experiment; (**b**) average plastic activity in the three grains of the cluster; the most active slip system is represented with a solid line and the second most active with a dashed line, while the grain color code is used.

Accumulated plasticity (all slip system contributions) within the bulk is shown in Figure 9a, and the slip activity for each of the three grains is detailed in Figure 9b. For Grains 4 and 18, which have close orientations and behave similarly, the model predicted double slip with systems (111)[0-11] and (1-11)[011], in agreement with the experimentally observed slip planes, and no other slip activity. The situation is different in Grain 10, which also showed double slip experimentally, but with planes (111) and (11-1), whereas the model predicts plastic activity on three planes (111), (1-11) and (11-1). It is interesting to see that despite the general agreement, the details of the slip system activation are far from perfect. The discrepancy may be attributed to the parameters of the constitutive laws, especially regarding the interaction matrix coefficients $h^{rs}$. These parameters were determined from macroscopic tensile curves and from the literature. A detailed parametric study is necessary to analyze the impact of these parameters values on the activation of slip systems in that grain. This can be seen as an opportunity to use such experimental data collected at the length scale of the grains and in the bulk to enrich identification datasets and solve the long-standing issue of crystal plasticity material parameter identification [39].

**Figure 10:**
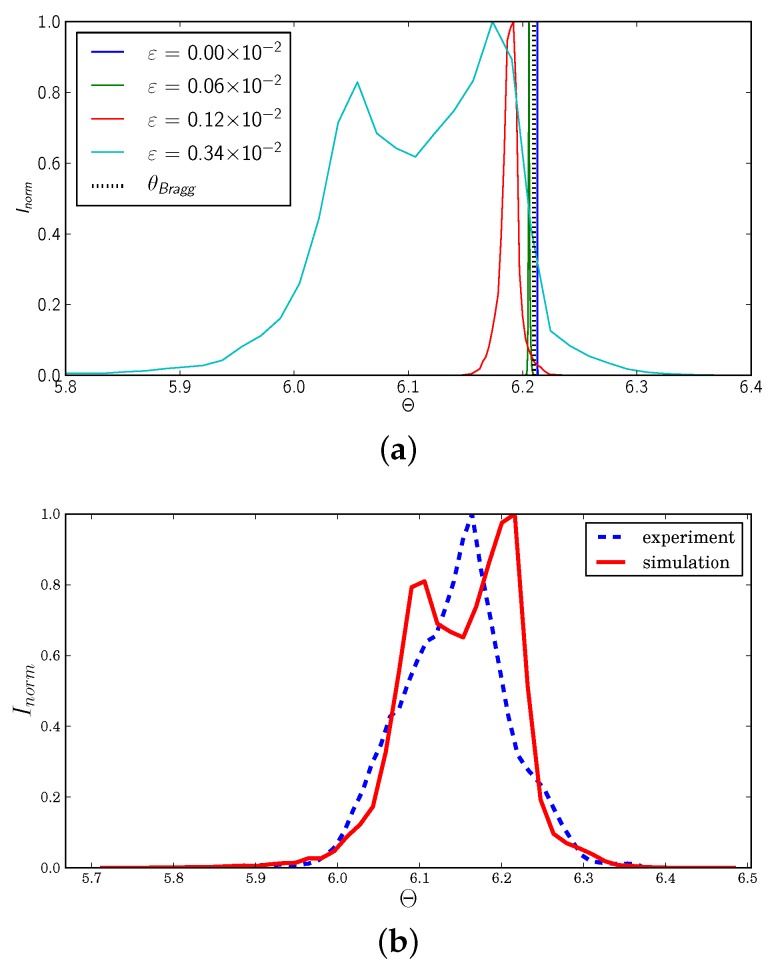
(**a**) Simulated rocking curves for Grain 4 at $\omega =165^{\circ }$ at four different strain levels; (**b**) comparison between experimental and simulated rocking curves at $\varepsilon =0.34\times 10^{-2}$.

Using the simulated mechanical fields within the grains, rocking curves can be simulated as described in Section 2.5. The generated rocking curves for each grain of the cluster are plotted in Figure 10, at $\omega =165^{\circ }$. The obtained behavior was consistent with the experimental observations. The rocking curve first shifted to lower Θ values due to the increase of the interplanar distance during loading. A very small amount of broadening due to the heterogeneous elastic strain field within the grain was also observed. When plasticity sets in and slip systems start to be active, the curve widening was more pronounced, and the shape changed, which can be related to the tendency to form subgrain-like regions. This effect had been studied both theoretically and experimentally, for instance by [40,41,42].

**Figure 11:**
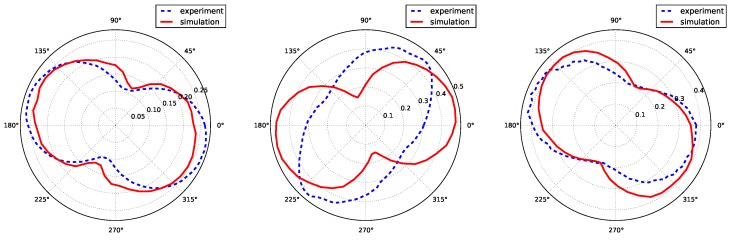
Comparison between experimentally simulated full width of the effective misorientation (FWEM) at $\varepsilon =0.34\times 10^{-2}$ for the three investigated grains.

Finally, a quantitative comparison between experimental FWEM and simulated FWEM is presented in Figure 11. Simulated curves are plotted for an applied strain of $\varepsilon_{33}=0.0034$, and experimental surfaces are plotted for the last step of the topotomography experiment. Both curves were in very good agreement for Grain 4 and Grain 18, for both shape and orientation. For Grain 10, the surface had the right amplitude, but not the right direction. As explained previously, the direction of the dumbbell-shaped curve was linked to the precise combination of active slip systems within the grain, and the discrepancy between the predicted and observed slip systems was presumably the cause of the mismatch in orientation for this case.

## 4. Conclusions

An in situ topotomographic experiment has been carried out at a synchrotron facility to collect a very detailed 4D dataset to study the onset of plastic slip under tension in an aluminum lithium alloy. Initial DCT imaging allowed measuring the initial microstructure and selecting a set of three grains in the bulk of the specimen for further investigations. Upon loading, incipient plasticity was observed non-destructively in the form of band contrast in X-ray topographs, which were further related geometrically to active slip planes in the bulk.

Information collected from rocking curves was presented in the form of polar plots illustrating the anisotropy of mean lattice curvature. The polar plots have a characteristic dumbbell shape, which can be attributed to lattice bending with respect to some specific axis, which could be determined in the experiment for the three grains during deformation.

Crystal plasticity simulations were carried out to compare the prediction obtained with a classical continuum model with our experimental observations. Essential features, such as slip system activation and average misorientation per grain, are well captured by the model, although some discrepancies remain for one grain. A major contribution of the work is the comparison of experimental and simulated misorientation polar plots showing characteristic dumbbell shapes quantifying the anisotropy of lattice curvature.

A striking feature of the presented experiment is the direct observation of inelastic deformation mechanisms in the bulk (plastic slip, but also twinning or phase transformation are possible). The detected slip system activity could be used in the material parameter identification procedure instead of macroscopic tests only. This would require a specific treatment (for instance using grain averaged quantities) since the full field calculation is costly and not well suited for an optimization routine.

One of the limitations of the present work is that only one specimen could be tested in the given beam-time. Building on this experiment and using the developed automation algorithms, future work will target more specimens and more grains per specimen to obtain more statistics and study slip transmission in more detail.

The adaptation of advanced reconstruction techniques [13] to this type of combined DCT and topotomography acquisitions might allow one to retrieve finer details of the orientation fields and their evolution at increasing levels of applied strain. This could be used to study more complex inelastic mechanisms in metals or to extract more dependable constitutive parameters minimizing the discrepancies between experimental observations and numerical simulations on the digital twins of the tested specimen.

## Figures and Tables

**Table 1:** Details of the 3 grains selected for TT imaging; the orientation convention is consistent with [28]; and angle values are given in ${}^{\circ }$.

Grain ID	Orientation (Rodrigues)	Aligned Reflection	$\theta_{\mathrm{Bragg}}$	(Φ,χ) Values ${(}^{\circ })$
4	[0.050, −0.305, 0.104]	(202)	6.21	(0.52, −11.04)
10	[−0.028, −0.145, 0.062]	(002)	4.39	(2.14, 16.63)
18	[−0.135, −0.272, −0.333]	(202)	6.21	(−4.81, −12.88)

**Table 2:** Material parameters identified from the macroscopic tensile tests.

*K* (MPa${}^{1/n}$)	*n* (-)	$\tau_{0}$ (MPa)	*Q* (MPa)	*b* (-)
38	10	10	5.3	763

**Table 3:** Edge-on ω values (in ${}^{\circ }$) for the two slip systems with the highest Schmid factor in the 3 grains; ω values in bold are used to show the topographs in edge-on configuration in Figure 5.

Grain ID	(hkl)	Schmid Factor	$\left(\omega_{1},\omega_{2}\right)$
4	(1-11)	0.476	(338.6, **176.3**)
	(111)	0.461	(158.6, 356.3)
10	(111)	0.490	(214.5, **40.8**)
	(1-11)	0.473	(304.5, 130.8)
18	(111)	0.488	(209.1, 46.8)
	(1-11)	0.457	(**29.1**, 226.8)

## Supplementary Materials

The following are available online at http://www.mdpi.com/1996-1944/11/10/2018/s1: Video S1: Full set of integrated topographs over 360 degrees (every $4^{\circ }$) for Grain 4 in the undeformed state; Video S2: Full set of integrated topographs over 360 degrees (every $4^{\circ }$) for Grain 4 in the deformed state.

## Abbreviations

The following abbreviations are used in this manuscript:

EDM	Electro-discharge machining
DCT	Diffraction contrast tomography
TT	Topotomography
PCT	Phase contrast tomography
CPFEM	Crystal plasticity finite element method
